# Cardiovascular and neuropsychiatric outcomes with PCSK9 inhibitors in atherosclerotic cardiovascular disease: A retrospective cohort study

**DOI:** 10.1002/bcp.70687

**Published:** 2026-07-07

**Authors:** Tianshu Gu, Yang Chen, Nan Zhang, Uazman Alam, Riccardo Proietti, Tong Liu, Kang‐Yin Chen, Gregory Y. H. Lip

**Affiliations:** ^1^ Liverpool Centre for Cardiovascular Science at University of Liverpool Liverpool John Moores University and Liverpool Heart and Chest Hospital Liverpool UK; ^2^ Tianjin Key Laboratory of Ionic‐Molecular Function of Cardiovascular disease, Department of Cardiology, Tianjin Institute of Cardiology The Second Hospital of Tianjin Medical University Tianjin China; ^3^ Department of Cardiovascular and Metabolic Medicine University of Liverpool Liverpool UK; ^4^ Liverpool Centre for Cardiovascular Sciences University of Liverpool and Liverpool University Hospitals NHS Foundation Trust Liverpool UK; ^5^ Centre for Biomechanics and Rehabilitation Technologies Staffordshire University Stoke‐on‐Trent UK; ^6^ Department of Clinical Medicine Aalborg University Aalborg Denmark; ^7^ Department of Cardiology, Lipidology and Internal Medicine with Intensive Coronary Care Unit Medical University of Bialystok Bialystok Poland

**Keywords:** atherosclerotic cardiovascular disease, neuropsychiatric disorders, PCSK9 inhibitors, real‐world evidence

## Abstract

**Aims:**

PCSK9 inhibitors reduce major cardiovascular events in randomized trials of atherosclerotic cardiovascular disease (ASCVD), but real‐world neuropsychiatric safety remains uncertain.

**Methods:**

In a propensity score–matched retrospective cohort study using the TriNetX U.S. Federated Research Network, adults with established ASCVD initiating alirocumab or evolocumab between 2017 and 2024 were matched 1:1 to statin‐monotherapy initiators. The 12‐month primary analysis assessed major adverse cardiovascular events (MACE: myocardial infarction, heart failure [HF], ischaemic stroke, all‐cause death), neurodegenerative disease (dementia, Parkinson's disease) and psychiatric disorders (psychosis, bipolar disorder, depression, anxiety), with Bonferroni correction and negative control. Secondary analyses examined individual agents, ASCVD subtypes, statin use, an intensified lipid‐lowering comparator and extended follow‐up.

**Results:**

Among 60 885 matched pairs (mean age 70 years; 61% male), PCSK9 inhibitor therapy was associated with lower 12‐month risks of MACE (HR 0.84, 95% CI 0.80–0.88), myocardial infarction (HR 0.82, 0.79–0.86), HF (HR 0.87, 0.83–0.92) and all‐cause mortality (HR 0.40, 0.37–0.43); ischaemic stroke did not differ. After Bonferroni correction, no neurodegenerative association remained significant. PCSK9 inhibitor use was associated with higher risks of any psychiatric diagnosis (HR 1.33, 1.25–1.42), depression (HR 1.44, 1.28–1.61) and anxiety (HR 1.39, 1.28–1.50), persisting in secondary analyses; negative control outcomes did not differ.

**Conclusions:**

PCSK9 inhibitor use was associated with favourable cardiovascular outcomes and no clear neurodegenerative harm, but higher rates of depression and anxiety. Given the observational design and potential residual confounding, statin‐intolerance‐related treatment selection and surveillance bias, these findings should be interpreted as hypothesis‐generating and warrant further prospective evaluation.

## Graphical abstract

Study design and main findings. After 1:1 propensity score matching, 60 885 PCSK9 inhibitor users and 60 885 statin‐only users with ASCVD were compared. Kaplan–Meier curves show 12‐month cumulative event‐free survival for MACE, psychiatric disease, and neurodegenerative disease. ASCVD, atherosclerotic cardiovascular disease; MACE, major adverse cardiovascular events; PCSK9i, PCSK9 inhibitor; PSM, propensity score matching. Schematic of study design and main findings: 1:1 propensity score–matched cohorts of 60 885 PCSK9 inhibitor users and 60 885 statin users with ASCVD, with Kaplan–Meier curves showing lower cardiovascular event rates, similar neurodegenerative outcomes, and higher psychiatric event rates in the PCSK9 inhibitor group.

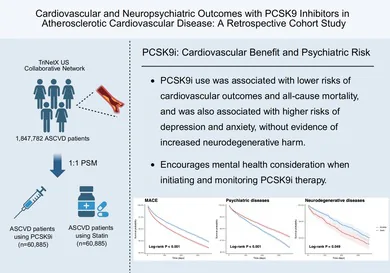

**What is already known about this subject**

- PCSK9 inhibitors lower LDL cholesterol and reduce major adverse cardiovascular events in randomized trials of patients with established atherosclerotic cardiovascular disease.
- Pharmacovigilance reports have raised the possibility of mood‐related adverse events with PCSK9 inhibitor use, but psychiatric outcomes have not been prespecified in cardiovascular outcome trials, and the magnitude of any risk in routine practice is uncertain.

**What this study adds**

- In a propensity score–matched cohort of 60 885 pairs of patients with atherosclerotic cardiovascular disease, PCSK9 inhibitor therapy was associated with lower 12‐month risks of major adverse cardiovascular events (HR 0.84, 95% CI 0.80–0.88), myocardial infarction (HR 0.82, 0.79–0.86), HF (HR 0.87, 0.83–0.92) and all‐cause mortality (HR 0.40, 0.37–0.43), with consistent estimates for alirocumab and evolocumab and through 36 months of follow‐up.
- PCSK9 inhibitor use was associated with a higher risk of depression (HR 1.44, 1.28–1.61) and anxiety (HR 1.39, 1.28–1.50) that persisted across individual agents, ASCVD subtypes, monotherapy *vs*. combination therapy, an intensified lipid‐lowering comparator, and extended follow‐up, while prespecified negative control outcomes showed no between‐group difference, supporting clinical vigilance for mood symptoms.

## 1 INTRODUCTION

Atherosclerotic cardiovascular disease (ASCVD) remains the leading cause of death and disability worldwide.^1^, ^2^ Elevated low‐density lipoprotein cholesterol (LDL‐C) is a major modifiable risk factor, and intensive lipid‐lowering therapy forms the cornerstone of secondary prevention.^3^ The proprotein convertase subtilisin/kexin type 9 (PCSK9) inhibitors have been established as highly potent lipid‐lowering agents that, when combined with statin therapy, can reduce LDL‐C levels by more than 60% and significantly lower the incidence of major adverse cardiovascular events (MACE).^4^, ^5^, ^6^, ^7^ Accordingly, current international lipid‐management guidelines recommend PCSK9 inhibitor for patients with ASCVD who do not achieve LDL‐C goals despite maximally tolerated statin therapy.^8^, ^9^

However, the potential effects of PCSK9 inhibition on brain health remain incompletely understood. Early observations have raised concerns that PCSK9 inhibitor use might be associated with an increased risk of neurocognitive adverse events.^10^ Nevertheless, findings from dedicated cognitive substudy data reported that PCSK9 inhibitor therapy did not result in significant decline in memory or executive function.^11^, ^12^, ^13^ A subsequent scientific statement from the American Heart Association underscored the lack of convincing evidence for neurocognitive harm but acknowledged that long‐term neurologic outcomes were uncertain.^14^ The association between PCSK9 inhibitor use and psychiatric disorders has also drawn attention. Cardiovascular outcome trials have not reported a consistent psychiatric safety signal, whereas pharmacovigilance analyses based on real‐world data have suggested increased depressive and mood‐related symptoms among PCSK9 inhibitor users.^15^, ^16^, ^17^, ^18^ Taken together, these findings suggest that further real‐world investigation is needed to better characterize the neuropsychiatric safety of PCSK9 inhibitors.

Given the relatively low incidence of neuropsychiatric outcomes in cardiovascular research, large‐scale real‐world data are required to enable adequately powered assessment of these associations. Against this background, we leveraged federated data from the TriNetX U.S. Federated Research Network to conduct a propensity score–matched (PSM) retrospective cohort study in patients with ASCVD. We aimed to examine associations between PCSK9 inhibitor therapy and three domains of outcomes^1^: MACE,^2^ neurodegenerative diseases and^3^ psychiatric disorders.

## 2 METHODS

### 2.1 Study design and data source

This was a retrospective cohort study conducted using the TriNetX US Federated Research Network (TriNetX LLC, Cambridge, MA, USA; https://trinetx.com), a federated health research platform providing access to de‐identified electronic health records (EHRs) from more than 72 health‐care organizations, mainly in the United States. The study was performed in accordance with the Strengthening the Reporting of Observational Studies in Epidemiology (STROBE) guidelines, and analyses were conducted on 27 September 2025.

All data undergo standardized quality control procedures within TriNetX based on conformance, completeness, and plausibility to ensure consistency and reliability. Available data elements include demographics, diagnoses (recorded using the International Classification of Diseases, Tenth Revision, Clinical Modification [ICD‐10‐CM]), procedures, medications, laboratory measurements and vital status. As all patient data within TriNetX are fully de‐identified in compliance with the Health Insurance Portability and Accountability Act (HIPAA) and General Data Protection Regulation (GDPR), institutional review board approval and informed consent were not required. We adopted a new‐user design, initiating follow‐up from the first recorded prescription to minimize immortal time bias. The TriNetX Research Network is fully compliant with HIPAA and holds a waiver from the Western Institutional Review Board, given the de‐identified and aggregate nature of the data. As this study used only de‐identified aggregate data under this waiver, a study‐specific ethics approval number was not applicable.

Additional details regarding the TriNetX data structure, governance and quality assurance are provided in the Appendix S1.

### 2.2 Study population

We identified adults (≥18 years) with established ASCVD from the TriNetX US Federated Research Network between 1 January 2017 (the year PCSK9 inhibitors were first approved in the United States) and 27 September 2024 (allowing at least 1 year of follow‐up for all patients). One group consisted of patients who received a PCSK9 inhibitor (alirocumab or evolocumab), either as monotherapy or in combination with a statin. The other group consisted of patients who received statin monotherapy. ASCVD was defined according to ICD‐10‐CM codes for ischaemic heart disease (IHD), cerebrovascular diseases or peripheral arterial disease (PAD). We excluded individuals with a documented diagnosis of neurodegenerative diseases (including dementia or Parkinson's disease) or psychiatric disorders (psychosis, bipolar disorder, depression or anxiety) before the index prescription date. The index date was uniformly defined as the first recorded prescription of PCSK9 inhibitor or statin, and baseline exclusions were applied symmetrically to both groups.

### 2.3 Outcomes

The study evaluated three prespecified categories of outcomes reflecting cardiovascular, neurodegenerative and psychiatric health domains.^1^ Cardiovascular outcomes included a composite endpoint of MACE defined as ischaemic stroke, acute myocardial infarction (AMI), heart failure (HF) and all‐cause death, as well as each component analysed separately.^2^ Neurodegenerative diseases comprised Alzheimer's disease, vascular dementia, dementia in other diseases classified elsewhere, unspecified dementia and Parkinson's disease.^3^ Psychiatric disorders encompassed psychosis, bipolar disorder, depression and anxiety disorders; these were additionally evaluated as a combined psychiatric composite outcome.

To assess potential residual confounding and validate the specificity of observed associations, we additionally evaluated a set of negative control outcomes (NCOs) unrelated to PCSK9 inhibitor exposure. This falsification analysis included otitis externa, folliculitis, kidney stones, gallbladder stones and burns, selected for their neutral pathophysiological relationship with lipid metabolism and cardiovascular disease. The composite NCO endpoint and individual events were analysed using the same PSM cohort framework and time‐to‐event approach. All outcomes were identified using standardized ICD‐10‐CM codes recorded in the TriNetX network (Table S1). Outcomes were evaluated as time‐to‐event endpoints, with prespecified reporting at 12 months (and additionally at 24 and 36 months).

### 2.4 Statistical analysis

Baseline characteristics were compared between groups using standardized mean differences (SMDs), with an SMD < 0.1 considered indicative of adequate balance. PSM was performed in a 1:1 ratio using greedy nearest‐neighbour matching with a calliper of 0.1 of the pooled standard deviation, as implemented within the TriNetX platform. The propensity score was derived from a logistic regression model, including demographic factors (age, sex, race/ethnicity), comorbidities (hypertension, IHD, diabetes mellitus, chronic kidney disease [CKD], obesity, HF, AMI), medications (cardiovascular procedures, beta blockers, lipid‐lowering agents, oral anticoagulants, angiotensin‐converting enzyme inhibitors [ACEIs], angiotensin receptor blockers [ARBs], ezetimibe, antidepressants, sedative/hypnotics) and patient services (Table 1). Treatment exposure was analysed according to an intention‐to‐treat approach, based on the initial therapy at index prescription, without reclassification for subsequent treatment changes.

**TABLE 1:** Baseline characteristics of ASCVD patients receiving PCSK9 inhibitor *vs* statin, before and after PSM.

Characteristics	Before PSM		SMD	After PSM		SMD
	PCSK9 inhibitor group (*n* = 61 003)	Statin group (*n* = 1 786 799)		PCSK9 inhibitor group (*n* = 60 885)	Statin group (*n* = 60 885)	
*Demographics*						
Current age	70.29 ± 10.13	73.38 ± 11.36	0.29	70.32 ± 10.12	70.31 ± 11.58	<0.01
Male	37 450 (61.39%)	1 160 580 (64.953%)	0.07	37 394 (61.417%)	37 373 (61.383%)	<0.01
Hispanic or Latino	2299 (3.769%)	115 437 (6.461%)	0.12	2297 (3.773%)	2160 (3.548%)	0.01
Asian	2302 (3.774%)	88 342 (4.944%)	0.06	2301 (3.779%)	2107 (3.461%)	0.02
White	47 123 (77.247%)	1 270 681 (71.115%)	0.14	47 012 (77.214%)	47 319 (77.719%)	0.01
Black or African American	5046 (8.272%)	239 810 (13.421%)	0.17	5045 (8.286%)	6081 (9.988%)	0.06
Nicotine dependence	4482 (7.347%)	205 830 (11.519%)	0.14	4479 (7.356%)	4645 (7.629%)	0.01
Alcohol related disorders	727 (1.192%)	46 775 (2.618%)	0.10	727 (1.194%)	826 (1.357%)	0.01
*Comorbidities*						
Hypertension	36 761 (60.261%)	1 209 597 (67.696%)	0.16	36 716 (60.304%)	34 401 (56.502%)	0.08
IHD	35 738 (58.584%)	702 214 (39.3%)	0.39	35 622 (58.507%)	34 166 (56.116%)	0.05
DM	15 692 (25.723%)	563 372 (31.53%)	0.13	15 677 (25.749%)	14 771 (24.26%)	0.03
Cerebrovascular diseases	9889 (16.211%)	347 660 (19.457%)	0.08	9875 (16.219%)	8897 (14.613%)	0.04
Heart failure	7581 (12.427%)	300 058 (16.793%)	0.12	7577 (12.445%)	6977 (11.459%)	0.03
AMI	7490 (12.278%)	192 378 (10.767%)	0.05	7475 (12.277%)	6845 (11.243%)	0.03
AF	7113 (11.66%)	299 337 (16.753%)	0.15	7111 (11.679%)	6490 (10.659%)	0.03
Atherosclerosis	4799 (7.867%)	130 580 (7.308%)	0.02	4794 (7.874%)	3573 (5.868%)	0.08
CKD	7024 (11.514%)	296 488 (16.593%)	0.15	7020 (11.53%)	6430 (10.561%)	0.03
Diseases of the respiratory system	18 483 (30.299%)	650 145 (36.386%)	0.13	18 457 (30.315%)	16 984 (27.895%)	0.05
Neoplasms	12 647 (20.732%)	416 219 (23.294%)	0.06	12 632 (20.747%)	11 728 (19.263%)	0.04
*Treatments*						
Therapeutic Cardiovascular services and procedures	5040 (8.262%)	83 091 (4.65%)	0.15	5002 (8.215%)	4575 (7.514%)	0.03
Hospital inpatient and observation care services	8805 (14.434%)	431 471 (24.148%)	0.25	8785 (14.429%)	9654 (15.856%)	0.04
Emergency department services	11 452 (18.773%)	507 805 (28.42%)	0.23	11 423 (18.762%)	12 054 (19.798%)	0.03
Office or other outpatient services	27 723 (45.445%)	828 987 (46.395%)	0.02	27 677 (45.458%)	25 916 (42.565%)	0.06
Percutaneous transluminal coronary angioplasty	558 (0.915%)	6874 (0.385%)	0.07	554 (0.91%)	391 (0.642%)	0.03
Analgesics	39 135 (64.153%)	1 322 943 (74.04%)	0.22	39 076 (64.18%)	36 566 (60.057%)	0.09
BBs	34 056 (55.827%)	1 049 676 (58.746%)	0.06	33 999 (55.841%)	32 065 (52.665%)	0.06
Anti‐platelets	33 112 (54.279%)	988 891 (55.344%)	0.02	33 041 (54.268%)	31 190 (51.228%)	0.06
Sedatives/hypnotics	22 786 (37.352%)	646 099 (36.16%)	0.02	22 722 (37.32%)	21 093 (34.644%)	0.06
Anaesthetics	22 353 (36.642%)	722 989 (40.463%)	0.08	22 306 (36.636%)	20 382 (33.476%)	0.07
Diuretics	21 393 (35.069%)	733 547 (41.054%)	0.12	21 367 (35.094%)	20 774 (34.12%)	0.02
Anticoagulants	21 368 (35.028%)	821 309 (45.965%)	0.22	21 328 (35.03%)	19 496 (32.021%)	0.06
CCBs	21 260 (34.851%)	670 772 (37.54%)	0.06	21 229 (34.867%)	18 932 (31.095%)	0.08
Ezetimibe	19 782 (32.428%)	69 692 (3.9%)	0.80	19 664 (32.297%)	18 167 (29.838%)	0.05
ARBs	18 288 (29.979%)	461 140 (25.808%)	0.09	18 254 (29.981%)	17 533 (28.797%)	0.03
ACEIs	15 220 (24.95%)	569 087 (31.85%)	0.15	15 196 (24.959%)	15 713 (25.808%)	0.02
*Patient services*						
Persons with potential health hazards related to socioeconomic and psychosocial circumstances	396 (0.649%)	21 950 (1.228%)	0.06	395 (0.649%)	370 (0.608%)	0.01
Problems related to housing and economic circumstances	131 (0.215%)	9713 (0.544%)	0.05	131 (0.215%)	158 (0.26%)	0.01
Problems related to education and literacy	21 (0.034%)	1443 (0.081%)	0.02	21 (0.034%)	23 (0.038%)	0.01
Problems related to certain psychosocial circumstances	10 (0.016%)	39 (0.002%)	0.01	10 (0.016%)	10 (0.016%)	0.01

*Note*: Therapeutic cardiovascular services and procedures refer to aggregated cardiovascular‐related procedures and services recorded in the TriNetX platform and were included to account for differences in baseline healthcare utilization and intensity of clinical contact between groups.

Abbreviations: ACEI, angiotensin‐converting enzyme inhibitor; AF, atrial fibrillation; ARB, angiotensin II receptor blocker; ASCVD, atherosclerotic cardiovascular disease; BBs, beta blockers; CCB, calcium channel blocker; CKD, chronic kidney disease; IHD, ischaemic heart disease; PAD, peripheral arterial disease; PSM, propensity score–matched; SMD, standardized mean difference.

Outcomes were analysed as time‐to‐event endpoints. Survival functions were estimated using the Kaplan–Meier method, with differences between groups assessed using log‐rank tests. Hazard ratios (HRs) with 95% confidence intervals (CIs) were calculated from Cox proportional hazards models. The proportional hazards assumption was evaluated using Schoenfeld residuals. Given the multiple outcomes assessed across cardiovascular, neurodegenerative and psychiatric domains, we applied Bonferroni correction to control for potential inflation of type I error because of multiple hypothesis testing. The corrected significance threshold was set at *P* < .0029, corresponding to .05 divided by 17 prespecified primary and secondary endpoints. Nominal *P*‐values were retained for descriptive interpretation, whereas Bonferroni‐adjusted thresholds were used to evaluate the robustness of statistical significance. The NCOs were not included in this correction procedure, as they were intended solely to assess residual confounding rather than to serve as inferential endpoints.

We also conducted five secondary analyses to examine the robustness of our findings. First, we separated PCSK9 inhibitors by individual agent, comparing alirocumab and evolocumab independently against statin therapy to assess potential drug‐specific differences in cardiovascular, neurodegenerative and psychiatric outcomes. Second, we analysed ASCVD subtypes including IHD, cerebrovascular disease and PAD to assess the consistency of associations across disease categories. Third, we stratified the PCSK9 inhibitor group by concomitant statin use, comparing PCSK9 inhibitor monotherapy and PCSK9 inhibitor–statin combination therapy separately against the statin‐only group. Fourth, we compared PCSK9 inhibitor therapy with a more intensive lipid‐lowering comparator comprising statin plus ezetimibe or bempedoic acid to assess whether the associations remained consistent when the control group more closely reflected the phenotype of PCSK9 inhibitors‐treated patients. Fifth, we explored outcomes at extended follow‐up periods of 24 months and 36 months to determine whether the observed associations persisted over longer durations of therapy.

All analyses were performed within the TriNetX platform (TriNetX LLC, Cambridge, MA) and R programming version 4.4.1 (The R Foundation for Statistical Computing, Vienna, Austria).

## 3 RESULTS

### 3.1 Characteristics of the study population

A total of 61 003 patients receiving PCSK9 inhibitors and 1 786 799 statin users with ASCVD were identified in the final cohort (Figure 1). PCSK9 inhibitors users were generally younger (mean age 70.3 ± 10.1 years *vs*. 73.4 ± 11.4 years) and had lower proportions of hypertension (60.3% *vs*. 67.7%), CKD (11.5% *vs*. 16.6%) and atrial fibrillation (11.7% *vs*. 16.8%), but a higher prevalence of IHD (58.6% *vs*. 39.3%). They were also more likely to receive ezetimibe (32.4% *vs*. 3.9%) and therapeutic cardiovascular procedures (8.3% *vs*. 4.7%). After 1:1 PSM, 60 885 matched pairs were included in the final analysis (Figure 1 and Table 1); all baseline characteristics were well balanced between groups (SMD < 0.1).

**FIGURE 1:**
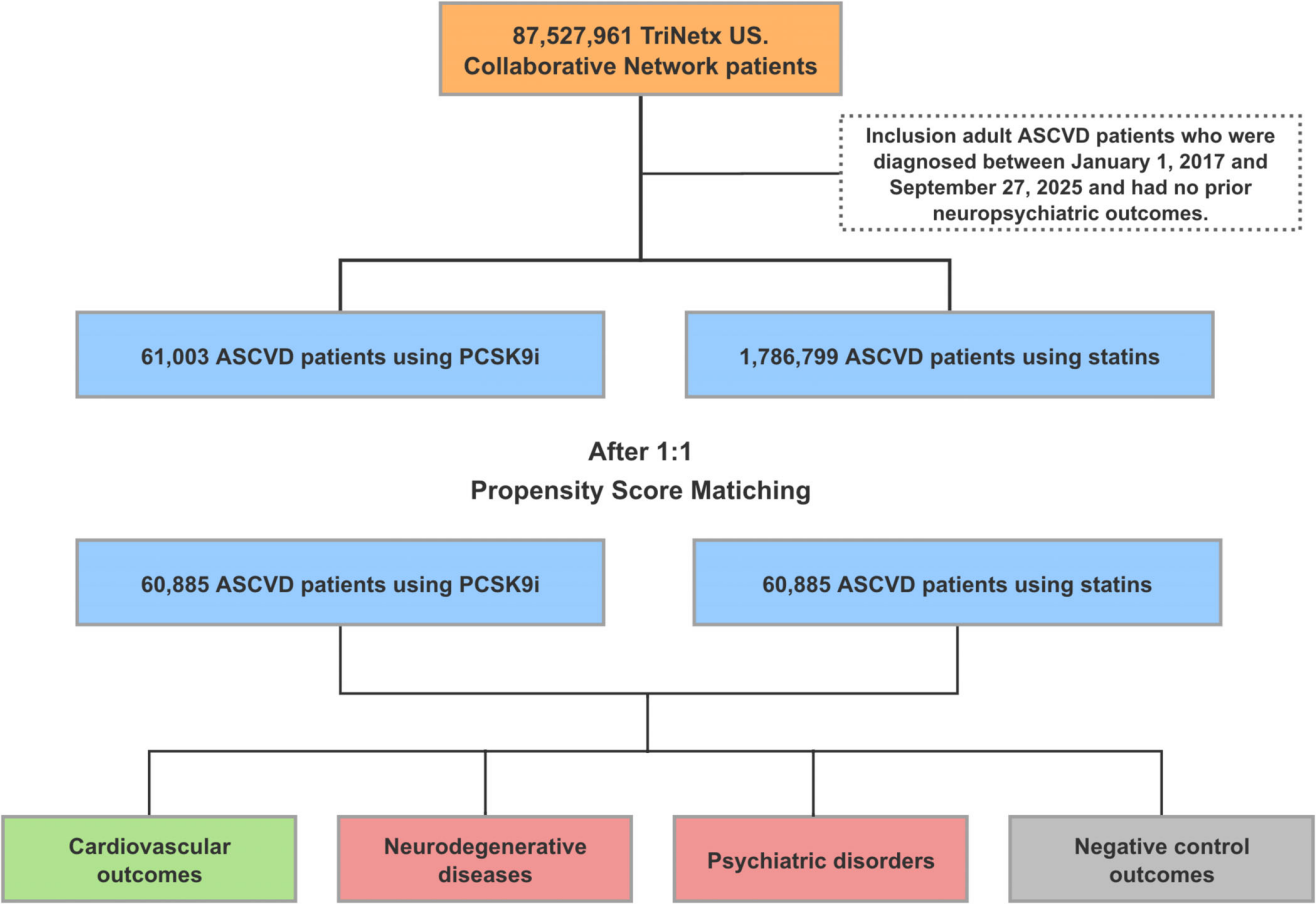
Workflow of this study. Flow diagram showing identification of 61 003 PCSK9 inhibitor users and 1 786 799 statin users with ASCVD from the TriNetX U.S. network, application of exclusion criteria and 1:1 propensity score matching that yielded 60 885 matched pairs for the primary analysis. ASCVD, atherosclerotic cardiovascular disease.

### 3.2 Primary analyses

At 12 months of follow‐up, PCSK9 inhibitor treatment was associated with a significantly lower risk of MACE compared with statins (7.68% *vs*. 8.99%; HR 0.84, 95% CI 0.80–0.88; Log rank *P* < .001) (Table 2 and Figure 2). All‐cause mortality was reduced in the PCSK9 inhibitor group (1.74% vs. 4.20%; HR 0.40, 95% CI 0.37–0.43; Log rank *P* < .001). The risk of AMI (4.42% *vs*. 5.02%; HR 0.82, 95% CI 0.79–0.86; Log rank *P* < .001) and HF (4.63% *vs*. 5.23%; HR 0.87, 95% CI 0.83–0.92; Log rank *P* < 0.001) was also significantly lower in PCSK9 inhibitors users. There was no significant difference in ischaemic stroke risk between groups (1.49% *vs*. 1.56%; HR 0.94, 95% CI 0.85–1.03; Log rank *P* = .190).

**TABLE 2:** One‐year outcomes in propensity score–matched ASCVD patients receiving PCSK9 inhibitors *vs.* statin therapy.

Outcome	Risk % (PCSK9 inhibitor)	Risk % (statin)	HR (95% CI)	*P* (log‐rank)
MACE	7.68%	8.99%	0.84 (0.80–0.88)	<.001**
Death	1.74%	4.20%	0.40 (0.37–0.43)	<.001**
Stroke	1.49%	1.56%	0.94 (0.85–1.03)	.190
AMI	4.42%	5.02%	0.82 (0.79–0.86)	<.001**
HF	4.63%	5.23%	0.87 (0.83–0.92)	<.001**
Neurodegenerative diseases	0.40%	0.48%	0.84 (0.70–0.99)	.049*
Dementia	0.32%	0.40%	0.78 (0.64–0.96)	.017*
Vascular dementia	0.07%	0.09%	0.75 (0.50–1.12)	.156
Dementia in other diseases	0.11%	0.10%	1.04 (0.73–1.46)	.843
Unspecified dementia	0.22%	0.27%	0.79 (0.63–0.99)	.039*
Alzheimer's disease	0.09%	0.09%	0.99 (0.68–1.45)	.974
Parkinson's disease	0.11%	0.10%	1.06 (0.74–1.53)	.752
Psychiatric diseases	3.62%	2.71%	1.33 (1.25–1.42)	<.001**
Psychosis	0.06%	0.07%	0.88 (0.56–1.40)	.590
Bipolar disorder	0.05%	0.05%	1.00 (0.58–1.71)	.988
Depression	1.39%	0.96%	1.44 (1.28–1.61)	<.001**
Anxiety disorder	2.69%	1.94%	1.39 (1.28–1.50)	<.001**

* For *P* < .05.

** For *P* < .0029 after Bonferroni correction.

Abbreviations: AMI, Acute myocardial infarction; ASCVD, atherosclerotic cardiovascular disease; CI, confidence interval; HF, heart failure; HR, hazard ratio; MACE, major adverse cardiovascular events.

**FIGURE 2:**
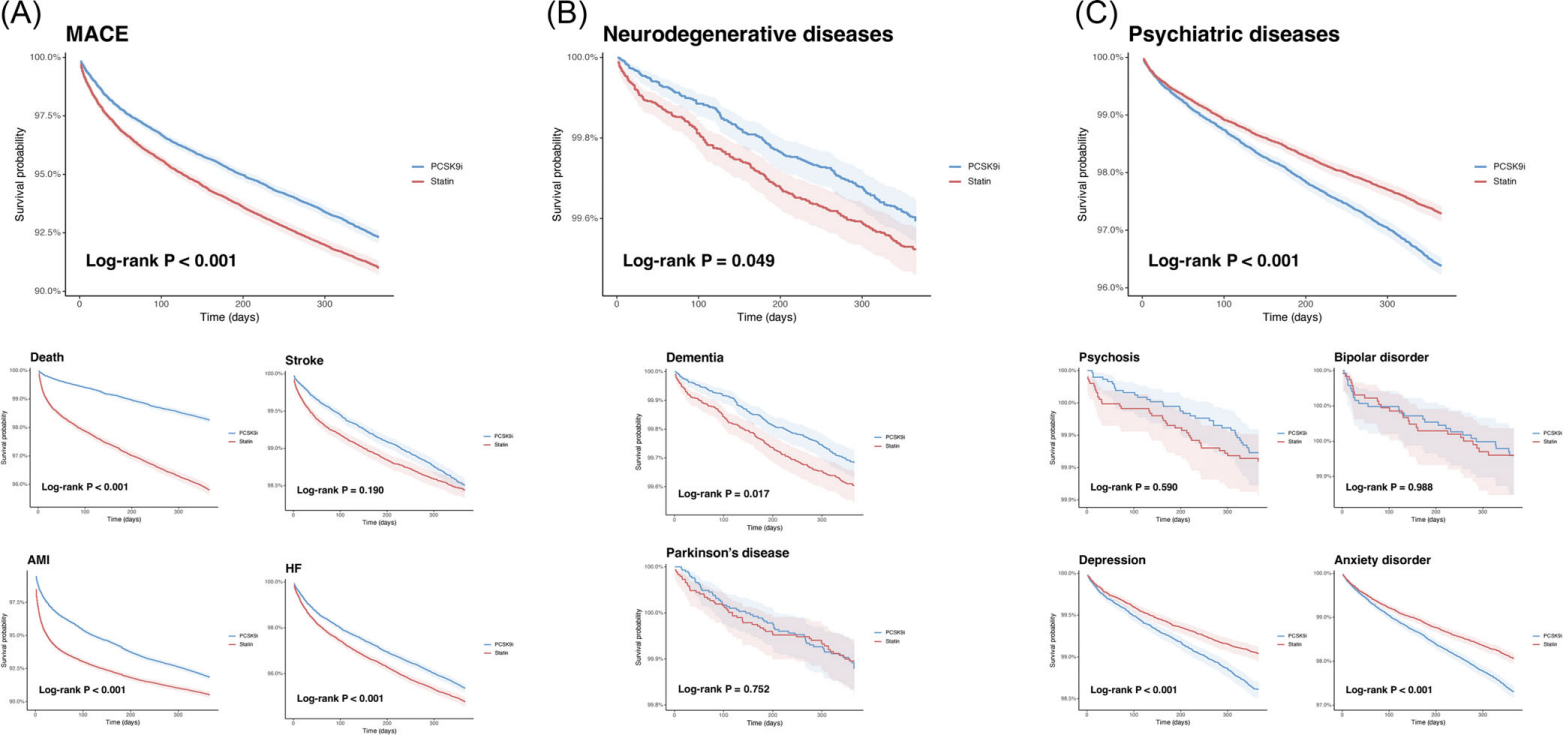
Twelve‐month cardiovascular and neuropsychiatric outcomes associated with PCSK9 inhibitors *vs.* statins. Kaplan–Meier survival curves comparing the 12‐month incidence of cardiovascular and neuropsychiatric outcomes between patients treated with PCSK9 inhibitors and statins after propensity‐score matching. (A) Major adverse cardiovascular events (MACE) and its components: all‐cause death, stroke, acute myocardial infarction (AMI) and heart failure (HF). (B) Neurodegenerative diseases and their subtypes: dementia and Parkinsons disease. (C) Psychiatric diseases and their subtypes: psychosis, bipolar disorder, depression and anxiety disorder. Shaded areas represent 95% confidence intervals, and *P*‐values are from log‐rank tests. Three panels of Kaplan–Meier survival curves comparing PCSK9 inhibitor and statin users over 12 months. Figure 2A shows higher cardiovascular event‐free survival with PCSK9 inhibitors (MACE and components); Figure 2B shows overlapping curves for neurodegenerative outcomes; and Figure 2C shows lower event‐free survival (higher event rates) with PCSK9 inhibitors for psychiatric outcomes.

Beyond cardiovascular endpoints, PCSK9 inhibitors use was associated with modest reductions in neurodegenerative diseases (0.40% *vs*. 0.48%; HR 0.84, 95% CI 0.70–0.99; Log rank *P* = .049) and dementia overall (0.32% *vs*. 0.40%; HR 0.78, 95% CI 0.64–0.96; Log rank *P* = .017), particularly unspecified dementia (0.22% *vs*. 0.27%; HR 0.79, 95% CI 0.63–0.99; Log rank *P* = .039). No significant differences were observed for vascular or Alzheimer's dementia or Parkinson's disease (Table 2).

Psychiatric disorders were more frequent among PCSK9 inhibitor users (3.62% *vs*. 2.71%; HR 1.33, 95% CI 1.25–1.42; Log rank *P* < .001), driven by higher rates of depression (1.39% *vs*. 0.96%; HR 1.44, 95% CI 1.28–1.61; Log rank *P* < .001) and anxiety disorder (2.69% *vs*. 1.94%; HR 1.39, 95% CI 1.28–1.50; Log rank *P* < .001).

When the Bonferroni correction was applied to account for multiple correlated outcomes across the cardiovascular, neurodegenerative and psychiatric domains, the associations of PCSK9 inhibitor use with MACE, AMI, HF and all‐cause mortality remained strongly significant (*P* < .0029). The modest reduction observed in overall neurodegenerative outcomes did not persist after adjustment. In contrast, the increased risks of depression and anxiety disorders among PCSK9 inhibitor users remained statistically significant even after Bonferroni correction (Figure 3).

**FIGURE 3:**
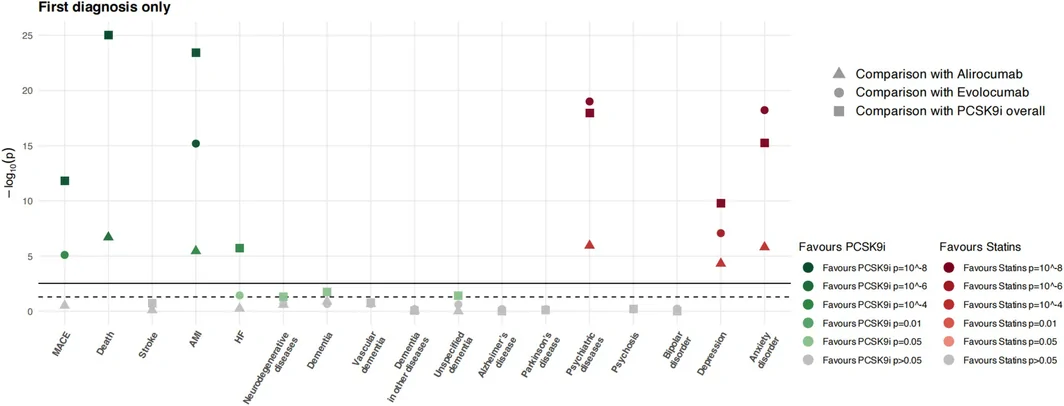
Association of PCSK9 inhibitors with cardiovascular and neuropsychiatric outcomes compared with statins. Statistical significance (−log₁₀[*P*]) of 1‐year associations between PCSK9 inhibitor therapy and cardiovascular, neurodegenerative, and psychiatric outcomes among patients with ASCVD after PSM. Triangles represent alirocumab, circles represent evolocumab, and squares represent the overall PCSK9 inhibitor group. The solid line denotes the Bonferroni‐corrected significance threshold (*P* < .0029), and the dashed line represents the nominal level (*P* < .05). Green tones indicate associations favouring PCSK9 inhibitors, and red tones indicate associations favouring statins. Plot of −log₁₀(*P*) values for each outcome, with triangles, circles and squares marking alirocumab, evolocumab and the overall PCSK9 inhibitor group. Associations for MACE, myocardial infarction, heart failure, mortality, depression, and anxiety cross the Bonferroni‐corrected threshold, whereas neurodegenerative associations do not. ASCVD, atherosclerotic cardiovascular disease; MACE, major adverse cardiovascular events.

### 3.3 Secondary analyses

In drug‐specific analyses (Appendix S2 and Tables S2–S3), evolocumab use was associated with a significantly lower risk of MACE (HR 0.86, 95% CI 0.81–0.92), AMI (HR 0.82, 95% CI 0.78–0.86) and all‐cause mortality (HR 0.43, 95% CI 0.39–0.47). Alirocumab showed similar reductions in AMI (HR 0.76, 95% CI 0.68–0.85) and death (HR 0.64, 95% CI 0.54–0.76), with a trend towards reduced MACE (HR 0.93, 95% CI 0.81–1.07). Neither agent significantly affected ischaemic stroke or HF risk.

For neurodegenerative outcomes, both drugs showed no significant association with dementia or Parkinson's disease. In contrast, both were linked to higher risks of psychiatric disorders, particularly depression and anxiety, with HRs of 1.57 (1.31–1.88) for alirocumab and 1.46 (1.34–1.58) for evolocumab (Appendix S2 and Tables S2–S3).

After Bonferroni correction for multiple comparisons, the associations of evolocumab with lower risks of MACE, HF, and all‐cause mortality and of alirocumab with lower mortality risk remained statistically significant (*P* < .0029). Both the overall PCSK9 inhibitor group and the individual agents showed no significant associations with any neurodegenerative outcomes, whereas higher risks of depression and anxiety persisted for both drugs (*P* < .0029) (Figure 3).

When stratified by ASCVD subtype (Appendix S2 and Table S4–S6), the associations between PCSK9 inhibitor therapy and clinical outcomes were largely consistent across vascular territories. For MACE and all‐cause mortality, PCSK9 inhibitor use conferred significant risk reductions in all three subgroups: IHD (MACE HR 0.82, 95% CI 0.78–0.87; death HR 0.40, 95% CI 0.37–0.43), cerebrovascular disease (HR 0.76, 95% CI 0.69–0.83; HR 0.42, 95% CI 0.38–0.47) and PAD (HR 0.82, 95% CI 0.75–0.90; HR 0.50, 95% CI 0.44–0.56). Regarding individual MACE components, PCSK9 inhibitor therapy was associated with lower risks of AMI (HR 0.74, 95% CI 0.71–0.77) and HF (HR 0.79, 95% CI 0.75–0.84) in the IHD subgroup, and with reduced AMI (HR 0.88, 95% CI 0.81–0.94) and stroke (HR 0.77, 95% CI 0.67–0.88) risks in the cerebrovascular subgroup. For neurodegenerative outcomes, PCSK9 inhibitor use was associated with modest lower risks of neurodegenerative diseases in both the IHD (HR 0.78, 95% CI 0.63–0.97) and cerebrovascular (HR 0.79, 95% CI 0.64–0.96) subgroups, primarily driven by decreased rates of unspecified dementia (IHD HR 0.77, 95% CI 0.60–0.99; cerebrovascular HR 0.69, 95% CI 0.53–0.90). No significant neurodegenerative risk reduction was observed in the PAD subgroup. For psychiatric outcomes, PCSK9 inhibitor use was consistently associated with higher risks across all ASCVD subgroups (IHD HR 1.28, 95% CI 1.19–1.37; cerebrovascular HR 1.10, 95% CI 1.00–1.22; PAD HR 1.30, 95% CI 1.16–1.44), mainly driven by elevated rates of depression and anxiety disorders (Appendix S2 and Table S4–S6).

Additionally, when stratifying PCSK9 inhibitor users by concomitant statin therapy (Appendix S2 and Table S7–S8), both PCSK9 inhibitor monotherapy and PCSK9 inhibitors–statin combination therapy were associated with lower all‐cause mortality compared with statin therapy alone, with greater reductions in cardiovascular events observed in the monotherapy subgroup. Neurodegenerative outcomes remained neutral, whereas the previously observed higher rates of depression and anxiety persisted in both groups.

In a sensitivity analysis comparing PCSK9 inhibitor therapy with a more intensive lipid‐lowering regimen of statin plus ezetimibe or bempedoic acid (Appendix S2 and Table S9), MACE showed no significant difference (HR 1.00, 95% CI 0.95–1.05). However, PCSK9 inhibitor use remained associated with lower 12‐month risks of all‐cause mortality (HR 0.59, 95% CI 0.54–0.63) and AMI (HR 0.93, 95% CI 0.89–0.97), no significant differences in dementia or other neurodegenerative outcomes, and persistently higher risks of depression (HR 1.44, 95% CI 1.29–1.61) and anxiety (HR 1.44, 95% CI 1.33–1.56).

With extended follow‐up (Appendix S2 and Tables S10–S11), the cardiovascular benefits of PCSK9 inhibitor therapy persisted over time, whereas associations with neuropsychiatric outcomes remained largely stable. At 24 months, PCSK9 inhibitor use was associated with lower risks of MACE (HR 0.90, 95% CI 0.87–0.94), AMI (HR 0.89, 95% CI 0.86–0.92), HF (HR 0.94, 95% CI 0.90–0.99) and all‐cause mortality (HR 0.50, 95% CI 0.47–0.53). Neurodegenerative outcomes were neutral, with no significant changes in any dementia subtype or Parkinson's disease. In contrast, psychiatric disorders remained more frequent among PCSK9 inhibitor users (HR 1.39, 95% CI 1.32–1.46), driven primarily by depression (HR 1.49, 95% CI 1.36–1.62) and anxiety (HR 1.47, 95% CI 1.38–1.57). At 36 months, these associations persisted (Appendix S2 and Table S11).

In the negative control outcome analysis (Appendix S2 and Table S12), there were no significant differences between PCSK9 inhibitor and statin users for the composite endpoint or any individual control event, including otitis externa, folliculitis, kidney stones, gallbladder stones and burns (*P* > .05 for all).

## 4 DISCUSSION

In this large, real‐world, PSM cohort of patients with ASCVD, our findings can be summarized in three principal observations. First, PCSK9 inhibitor therapy was associated with consistent relative risk reductions across cardiovascular endpoints, with lower risks of MACE, AMI, HF and all‐cause mortality. Second, the use of PCSK9 inhibitors was associated with higher rates of psychiatric disorders, particularly depression and anxiety, and these associations remained significant after Bonferroni correction. Third, although modest reductions in neurodegenerative outcomes were observed, these associations were no longer significant after adjustment, indicating that PCSK9 inhibitor therapy was not significantly associated with neurocognitive outcomes.

The consistency of these findings was further demonstrated across alirocumab and evolocumab, across ASCVD subgroups, across PCSK9 inhibitor monotherapy compared with PCSK9 inhibitor plus statin therapy, across comparisons with an intensified control group receiving statin plus ezetimibe or bempedoic acid, and across extended follow‐up periods of up to 36 months. The NCOs analysis further supported the robustness and specificity of these findings. Collectively, these results suggest a complex clinical profile of PCSK9 inhibition in real‐world practice, characterized by consistent cardiovascular risk reduction, no clear evidence of neurocognitive harm and an association with higher rates of psychiatric morbidity.

Our findings are broadly consistent with evidence from randomized trials and genetic studies demonstrating cardiovascular benefit and no convincing neurocognitive harm with PCSK9 inhibition, while additionally identifying a psychiatric association that has been difficult to quantify in controlled trial settings. Pooled evidence from large‐scale meta‐analyses of alirocumab and evolocumab consistently indicates that PCSK9 inhibitors significantly reduce the risk of major cardiovascular events without additional hepatic, muscular, or neurocognitive toxicity.^6^, ^19^, ^20^ A recent network meta‐analysis further extended this evidence by comparing different PCSK9 inhibitor regimens in patients with established ASCVD.^21^ Observational studies have also suggested lower all‐cause mortality among PCSK9 inhibitor users.^22^ Building upon this existing evidence, our study examined these associations in a large, heterogeneous real‐world cohort of patients with ASCVD, including those with IHD, cerebrovascular disease and PAD. The magnitude of the all‐cause mortality reduction observed in the present cohort (HR 0.40) notably exceeds effect sizes reported in controlled trial settings, where mortality benefits of PCSK9 inhibitors have generally been more modest. While the large, multicentre TriNetX U.S. Federated Research Network provided substantial statistical power and captured a heterogeneous routine‐care population, residual confounding and treatment‐selection bias remain possible. Patients receiving PCSK9 inhibitors in routine practice may have greater access to specialist lipid care, closer follow‐up, better adherence to preventive therapy and different baseline risk profiles compared with statin‐only users. Unmeasured frailty, disease severity, LDL‐C levels, treatment targets, socioeconomic factors and reasons for treatment escalation could also have contributed to the discrepancy. Therefore, the mortality reduction should be regarded as a real‐world association observation rather than a direct estimate of causal mortality benefit.

Our real‐world findings regarding neurocognitive outcomes are consistent with previous randomized trials and post hoc analyses evaluating the safety of PCSK9 inhibitors. In the ODYSSEY OUTCOMES, CANTAB and FOURIER studies, patients receiving PCSK9 inhibitors showed no evidence of cognitive impairment, even at very low LDL‐C levels.^13^, ^23^, ^24^ Genetic and Mendelian randomization studies provide additional reassurance that lifelong modulation of PCSK9 or HMGCR activity did not increase the risk of Alzheimer's disease, vascular dementia, or Parkinson's disease.^25^, ^26^

A large‐scale Mendelian randomization analysis reported that genetically proxied cholesterol lowering via HMGCR, NPC1L1 and CETP pathways, but not through PCSK9, was associated with a lower long‐term risk of all‐cause dementia.^27^ This aligns with our observation that, after Bonferroni correction, PCSK9 inhibitor use was not significantly associated with neurodegenerative outcomes. An earlier meta‐analysis reported an increased incidence of neurocognitive adverse events; however, these findings were derived primarily from spontaneous adverse event reporting in cardiovascular outcome trials, where neurocognitive outcomes were not prespecified and event ascertainment was heterogeneous.^10^ Subsequent evidence, including the American Heart Association scientific statement on aggressive LDL‐C lowering, concluded that PCSK9 inhibitors and other intensive lipid‐lowering therapies are not associated with convincing neurocognitive toxicity.^14^ Our study contributes complementary real‐world evidence showing no detectable association between PCSK9 inhibitor use and neurocognitive outcomes within the available follow‐up period.

A unique observation of this study is the consistently higher risk of depression and anxiety associated with PCSK9 inhibitor use, observed across different agents, ASCVD subtypes and extended follow‐up periods. Recent pharmacovigilance investigations have likewise reported excess neuropsychiatric and mood‐related adverse events among PCSK9 inhibitor users, particularly depressive symptoms emerging during treatment.^17^, ^18^, ^28^ Genetic analyses of the PCSK9 locus further linked LDL‐lowering alleles with mood instability and neuroticism, suggesting biologically plausible pleiotropic mechanisms.^16^, ^29^ Collectively, these findings suggest that PCSK9 inhibition may be associated with differential effects across cardiovascular and psychiatric domains, underscoring the importance of individualized risk–benefit assessment and clinical vigilance.

Several interpretive considerations apply specifically to the psychiatric findings. First, residual confounding by treatment indication cannot be excluded: PCSK9 inhibitors are generally prescribed to patients with more severe dyslipidaemia, insufficient LDL‐C goal attainment despite maximally tolerated lipid‐lowering therapy, or higher perceived cardiovascular risk, and these factors could not be directly incorporated into the propensity score model because complete lipid laboratory data, LDL‐C trajectories, and reasons for PCSK9 inhibitor initiation were not consistently available in the TriNetX platform. Second, statin intolerance—one of the common reasons for initiating PCSK9 inhibitor therapy—represents an important additional source of bias, as statin‐associated symptoms, treatment discontinuation and nonadherence have been associated with depression and anxiety.^30^, ^31^, ^32^ Statin intolerance was likewise not available as a consistently captured structured variable in TriNetX and therefore could not be incorporated into the matching process. Third, surveillance and detection biases may have contributed to higher recorded rates of psychiatric diagnoses, given that patients receiving PCSK9 inhibitors may have more frequent contact with lipid clinics, cardiologists, or other specialist services, increasing the opportunity for mood symptoms to be recognized and coded. To mitigate these concerns, the propensity score model incorporated a broad range of cardiovascular comorbidities, lipid‐lowering and cardiovascular medications, antidepressant and sedative/hypnotic use, and healthcare‐utilization variables; sensitivity analyses using an intensified lipid‐lowering comparator group and analyses stratified by concomitant statin use showed broadly consistent psychiatric associations across subgroups; and prespecified negative control outcomes showed no significant between‐group differences, arguing against a generalized increase in coded diagnoses among PCSK9 inhibitor users. Nevertheless, these measures cannot fully exclude residual confounding from unmeasured statin intolerance, LDL‐C levels, disease severity, psychological distress related to high‐risk cardiovascular status or differential ascertainment of psychiatric diagnoses. Therefore, the higher recorded rates of depression and anxiety should be interpreted as hypothesis‐generating associations that warrant prospective confirmation, rather than as evidence of a direct pharmacological effect.

The characteristic pattern observed in this study—marked cardiovascular benefit, neutral or mildly protective neurocognitive effects and higher recorded rates of psychiatric diagnoses among PCSK9 inhibitor users—may reflect two underlying biological pathways: one mediated by low‐density lipoprotein cholesterol (LDL‐C) reduction and the other involving PCSK9‐specific mechanisms beyond lipid lowering. The LDL‐C–mediated pathway directly explains the cardiovascular benefits and neurocognitive neutrality. Substantial LDL‐C reduction decreases atherothrombotic burden, stabilizes vulnerable plaques and lowers the incidence of ischaemic events, findings that align with the observed reductions in MACE, AMI and mortality. However, within the brain, cholesterol metabolism is largely autonomous. The blood–brain barrier restricts exchange between peripheral and central cholesterol pools, and cerebrospinal fluid PCSK9 concentrations remain stable even when plasma levels fluctuate.^33^ In contrast, PCSK9‐specific biological functions independent of lipid lowering could represent one possible explanation for the psychiatric associations observed in this study, although residual confounding and treatment‐selection bias remain plausible alternative explanations. A large Mendelian randomization analysis found that genetically proxied PCSK9 inhibition was associated with a higher risk of depression, representing a drug target–specific signal not explained by statin therapy or overall lipid lowering.^34^ Complementary evidence from the UK Biobank showed that LDL‐lowering PCSK9 alleles were related to mood instability and neuroticism, suggesting shared pathways between metabolic and affective regulation.^16^ Mechanistically, PCSK9 is expressed in neurons, astrocytes and microglia, where it contributes to receptor trafficking, synaptic remodelling and inflammatory signalling. Although the blood–brain barrier limits peripheral–central exchange under physiological conditions, PCSK9 expression within the central nervous system can change dynamically in disease states, potentially influencing affective neural circuits.^33^

In summary, our study observed associations between PCSK9 inhibitor use, favourable cardiovascular outcomes, generally neutral neurocognitive findings, and higher recorded rates of depression and anxiety. These psychiatric findings should be interpreted cautiously given the observational design and the potential for residual confounding, treatment‐selection bias and differential outcome ascertainment. Future prospective studies with dedicated neuropsychiatric assessments and mechanistic investigations are needed to determine whether these associations reflect direct effects of PCSK9 inhibition or characteristics of the underlying patient population.

## 5 LIMITATIONS

Our study has several limitations. First, as an observational retrospective cohort based on the TriNetX EHR‐derived network, residual confounding, coding inaccuracies, and potential misclassification or under‐ascertainment of psychiatric and neurodegenerative outcomes identified using ICD‐10 codes cannot be excluded. Differential health‐care contact may also have influenced the likelihood of psychiatric diagnosis recording. Second, follow‐up was limited to a maximum of 36 months, which may not capture very late‐onset neurocognitive or psychiatric effects. Third, although we analysed two PCSK9 inhibitors (alirocumab and evolocumab), data on inclisiran were not included because of its more recent market entry and zero users in our matched cohort; we excluded all inclisiran patients. Fourth, for several small population, secondary analyses in the PCSK9 inhibitor–statin combination subgroup, the TriNetX platform returned a lack of results because of the small numbers. Even so, the key outcomes of interest remained fully analysable, such as MACE, depression and anxiety. Fifth, complete lipid laboratory data, LDL‐C trajectories, LDL‐C goal attainment, treatment adherence, statin intolerance, statin‐associated symptoms and reasons for PCSK9 inhibitor initiation were not consistently available. Given that PCSK9 inhibitors are commonly prescribed to patients with more severe dyslipidaemia, insufficient LDL‐C control or statin intolerance, residual confounding by indication cannot be excluded. Although propensity score matching adjusted for multiple relevant variables and several sensitivity analyses were performed, unmeasured treatment‐selection factors may still have influenced both cardiovascular and psychiatric outcomes. Therefore, the psychiatric associations should be considered hypothesis‐generating.

## 6 CONCLUSION

PCSK9 inhibitor use was associated with favourable cardiovascular outcomes and no clear evidence of neurocognitive harm; however, it was also associated with higher risks of depression and anxiety. These findings support clinical awareness and follow‐up monitoring for mood‐related symptoms among PCSK9 inhibitor users. These psychiatric associations should be interpreted as hypothesis‐generating given the observational design and potential residual confounding and surveillance bias. Prospective studies with dedicated neuropsychiatric assessments and mechanistic investigations are needed to confirm these findings and clarify their underlying mechanisms.

## AUTHOR CONTRIBUTIONS

Gregory Y. H. Lip conceived the study and supervised all aspects of the work. Tianshu Gu performed the primary analyses and drafted the manuscript. Nan Zhang and Yang Chen assisted with operation of the TriNetX platform. Tong Liu and Kang‐Yin Chen provided clinical oversight to study design and outcomes. Uazman Alam and Riccardo Proietti contributed to data interpretation and methodological guidance. Yang Chen and Nan Zhang critically revised the manuscript.

## CONFLICT OF INTEREST STATEMENT

G.Y.H.L. reports institutional consultancy and/or speaker roles for BMS/Pfizer, Boehringer Ingelheim, Anthos and Huawei (No fees are received personally.). He is co‐PI of the AFFIRMO project on multimorbidity in AF (grant agreement No 899871), TARGET project on digital twins for personalized management of atrial fibrillation and stroke (grant agreement No 101136244) and ARISTOTELES project on artificial intelligence for management of chronic long‐term conditions (grant agreement No 101080189), which are all funded by the EU's Horizon Europe Research & Innovation programme. Others: None declared.

## Supporting information


**Data S1.** Supporting Information.

**Appendix S1.**Supplementary Methods.
**Appendix S2.** Supplemental Tables.

## Data Availability

The analyses were conducted using the TriNetX U.S. Federated Research Network, which provides access to de‐identified patient‐level data within a federated analytics environment. Per TriNetX data governance policies, the raw underlying data cannot be directly shared externally. However, institutions with TriNetX platform access can reproduce the analyses independently within the platform. The data that support the findings of this study are available from TriNetX LLC. Restrictions apply to the availability of these data, which were used under license for this study. Data are available from https://trinetx.com with the permission of TriNetX LLC.
