# Supplementary material for: Cardiovascular and neuropsychiatric outcomes with PCSK9 inhibitors in atherosclerotic cardiovascular disease: A retrospective cohort study
Source: Br J Clin Pharmacol. 2026 Jul 7;92(11):3862–73. doi: 10.1002/bcp.70687 (PMC13714615; doi:10.1002/bcp.70687)
Supplement: Supplementary file 1 [file BCP-92-3862-s001.docx]

**Cardiovascular and Neuropsychiatric Outcomes with PCSK9 Inhibitors in Atherosclerotic Cardiovascular Disease: A Retrospective Cohort Study**

**Supplementary material**

Tianshu Gu, Yang Chen, Nan Zhang, Uazman Alam, Riccardo Proietti, Tong Liu, Kang-Yin Chen, Gregory Y. H. Lip

**Supplemental Notes**

| **Appendix S1.** | Supplementary Methods |  |
| --- | --- | --- |

| **Appendix S2.** | Supplemental Tables |  |
| --- | --- | --- |
| **eTable1.** | ICD-10-CM, VA, and RxNorm codes used for comorbidities, medications, and study outcomes |  |
| **eTable2.** | One-year outcomes of evolocumab versus statins in the ASCVD population |  |
| **eTable3.** | One-year outcomes of alirocumab versus statins in the ASCVD population |  |
| **eTable4.** | One-year outcomes of PCSK9i versus statins in patients with ischemic heart disease |  |
| **eTable5.** | One-year outcomes of PCSK9i versus statins in patients with Cerebrovascular disease |  |
| **eTable6.** | One-year outcomes of PCSK9i versus statins in patients with peripheral arterial disease |  |
| **eTable7.** | 12-month outcomes of PCSK9i–statin combination therapy versus statin therapy in patients with ASCVD |  |
| **eTable8.** | 12-month outcomes of PCSK9i monotherapy versus statin therapy in patients with ASCVD |  |
| **eTable9.** | 12-month outcomes of PCSK9i versus statin plus ezetimibe or bempedoic acid therapy in patients with ASCVD |  |
| **eTable10.** | 24-month outcomes of PCSK9i versus statins in patients with ASCVD |  |
| **eTable11.** | 36-month outcomes of PCSK9i versus statins in patients with ASCVD |  |
| **eTable12.** | 12-month negative control outcomes of PCSK9i versus statins in patients with ASCVD |  |

#### **Appendix S1. Supplementary Methods**

## The TriNetX U.S. Federated Research Network (TriNetX LLC, Cambridge, MA, USA) is a federated health research platform that provides access to de-identified electronic health records from participating healthcare organizations, mainly in the United States. Each healthcare organization (HCO) maintains its own local data repository, referred to as a TriNetX “appliance”, which is physically hosted at the institution’s data center or on a secure virtual server. When a query is executed on the TriNetX platform, it is broadcast across these appliances, and only aggregated, de-identified results are returned to the user in real time. Within the network, data are harmonized to a standardized data model and mapped to common clinical terminologies, including ICD-10-CM for diagnoses, ICD-10-PCS and Current Procedural Terminology (CPT) for procedures, Logical Observation Identifiers Names and Codes (LOINC) for laboratory results, and RxNorm for medications. This federated structure allows analyses to be performed without direct transfer of patient-level identifiable data and supports the assessment of real-world clinical populations across participating healthcare organizations.

## HIPAA and GDPR Compliance. All TriNetX data are de-identified according to the Health Insurance Portability and Accountability Act (HIPAA) Privacy Rule standard for de-identification and are further reviewed under a standing waiver granted by the Western Institutional Review Board (WIRB). This process ensures that no protected health information (PHI) or personal identifiers are transmitted outside the source institution. TriNetX retains only aggregated counts and statistical summaries that cannot be used to re-identify individual patients. In addition, TriNetX and its participating HCOs operate under applicable data privacy and local jurisdictional policies governing data handling. As a result, studies using the TriNetX network are generally exempt from institutional review board approval and patient informed consent because no individually identifiable information is accessible to researchers. Further information about the TriNetX data model, data governance, and quality assurance framework is available at https://trinetx.com.

#### **Appendix S2. Supplemental Tables.**

**eTable1. ICD-10-CM, VA, and RxNorm codes used for comorbidities, medications, and study outcomes**

| ICD-10-CM / VA / RxNorm codes (TriNetX definition) | Definition / Variable |
| --- | --- |
| 2106-3 | White |
| 2186-5 | Not Hispanic or Latino |
| 2135-2 | Hispanic or Latino |
| 2054-5 | Black or African American |
| 2028-9 | Asian |
| I25 | Chronic ischemic heart disease |
| I21 | Acute myocardial infarction |
| I10-I1A | Hypertensive diseases |
| I50 | Heart failure |
| I48 | Atrial fibrillation and flutter |
| E08-E13 | Diabetes mellitus |
| E65-E68 | Overweight, obesity and other hyperalimentation |
| G00-G99 | Diseases of the nervous system |
| N18 | Chronic kidney disease (CKD) |
| J00-J99 | Diseases of the respiratory system |
| F01-F99 | Mental, Behavioral and Neurodevelopmental disorders |
| C00-D49 | Neoplasms |
| D50-D89 | Diseases of the blood and blood-forming organs and certain disorders involving the immune mechanism |
| Z55-Z65 | Persons with potential health hazards related to socioeconomic and psychosocial circumstances |
| Z40-Z53 | Encounters for other specific health care |
| Z00-Z13 | Persons encountering health services for examinations |
| Z69-Z76 | Persons encountering health services in other circumstances |
| Z59 | Problems related to housing and economic circumstances |
| Z63 | Other problems related to primary support group, including family circumstances |
| Z64 | Problems related to certain psychosocial circumstances |
| Z62 | Problems related to upbringing |
| Z55 | Problems related to education and literacy |
| Z57 | Occupational exposure to risk factors |
| Z56 | Problems related to employment and unemployment |
| Z60 | Problems related to social environment |
| Z72.0 | Tobacco use |
| F10 | Alcohol related disorders |
| F17 | Nicotine dependence |
| I73 | Other peripheral vascular diseases |
| I70 | Atherosclerosis |
| I77 | Other disorders of arteries and arterioles |
| 1021163 | Percutaneous transluminal coronary angioplasty |
| 1021165 | Percutaneous transcatheter placement of intracoronary stent(s), with coronary angioplasty when performed |
| 1012975 | Therapeutic Cardiovascular Services and Procedures |
| 1013626 | Office or Other Outpatient Services |
| 1013659 | Hospital Inpatient and Observation Care Services |
| 1013711 | Emergency Department Services |
| CV100 | BETA BLOCKERS/RELATED |
| CV700 | DIURETICS |
| CV200 | CALCIUM CHANNEL BLOCKERS |
| CV805 | ANGIOTENSIN II INHIBITOR |
| CV800 | ACE INHIBITORS |
| BL117 | PLATELET AGGREGATION INHIBITORS |
| BL110 | ANTICOAGULANTS |
| AN000 | ANTINEOPLASTICS |
| IM000 | IMMUNOLOGICAL AGENTS |
| CN100 | ANALGESICS |
| CN300 | SEDATIVES/HYPONTICS |
| CN600 | ANTIDEPRESSANTS |
| CN200 | ANESTHETICS |
| HS502 | ORAL HYPOGLYCEMIC AGENTS, ORAL |
| 341248 | ezetimibe |
| 83367 | atorvastatin |
| 301542 | Rosuvastatin |
| H60 | Otitis externa |
| L73 | Folliculitis |
| N20.0-N20.2 | Kidney stones |
| K80 | Gallbladder stones |
| T20-T32 | Burns |

**eTable2. 12-month outcomes of evolocumab versus statins in the ASCVD population**

| Outcome | Risk % (Evolocumab) | Risk % (Statin) | HR (95 CI) | p (Log-rank) |
| --- | --- | --- | --- | --- |
| MACE | 7.77 | 8.82 | 0.86 (0.81-0.92) | **<0.001** |
| Death | 1.83 | 4.16 | 0.43 (0.39-0.47) | **<0.001** |
| Stroke | 1.48 | 1.55 | 0.93 (0.82-1.06) | 0.264 |
| AMI | 4.13 | 4.91 | 0.82 (0.78-0.86) | **<0.001** |
| HF | 4.78 | 5.08 | 0.93 (0.86-1.00) | 0.036 |
| Neurodegenerative diseases | 0.45 | 0.50 | 0.87 (0.70-1.08) | 0.219 |
| Dementia | 0.36 | 0.41 | 0.86 (0.68-1.09) | 0.213 |
| Vascular dementia | 0.09 | 0.06 | 1.41 (0.82-2.42) | 0.216 |
| Dementia in other diseases | 0.12 | 0.12 | 0.90 (0.59-1.38) | 0.635 |
| Unspecified dementia | 0.25 | 0.29 | 0.84 (0.63-1.12) | 0.239 |
| Alzheimer’s disease | 0.10 | 0.08 | 1.13 (0.69-1.84) | 0.624 |
| Parkinson’s disease | 0.11 | 0.12 | 0.90 (0.58-1.39) | 0.637 |
| Psychiatric diseases | 4.04 | 2.75 | 1.46 (1.34-1.58) | **<0.001** |
| Psychosis | 0.06 | 0.07 | 0.89 (0.51-1.57) | 0.687 |
| Bipolar disorder | 0.05 | 0.06 | 0.83 (0.44-1.56) | 0.568 |
| Depression | 1.55 | 1.08 | 1.43 (1.25-1.63) | **<0.001** |
| Anxiety disorder | 3.03 | 1.95 | 1.54 (1.40-1.70) | **<0.001** |

#### **eTable3. 12-month outcomes of alirocumab versus statins in the ASCVD population**

| Outcome | Risk % (Alirocumab) | Risk % (Statin) | HR (95 CI) | p (Log-rank) |
| --- | --- | --- | --- | --- |
| MACE | 9.01 | 9.60 | 0.93 (0.81-1.07) | 0.306 |
| Death | 3.21 | 4.86 | 0.64 (0.54-0.76) | **<0.001** |
| Stroke | 1.77 | 1.80 | 0.96 (0.74-1.26) | 0.784 |
| AMI | 5.12 | 5.58 | 0.76 (0.68-0.85) | **<0.001** |
| HF | 5.01 | 5.21 | 0.95 (0.81-1.12) | 0.563 |
| Neurodegenerative diseases | 0.53 | 0.68 | 0.78 (0.50-1.20) | 0.251 |
| Dementia | 0.40 | 0.58 | 0.69 (0.42-1.12) | 0.129 |
| Vascular dementia | 0.06 | 0.09 | 1.41 (0.82-2.42) | 0.216 |
| Dementia in other diseases | 0.18 | 0.19 | 0.91 (0.42-2.00) | 0.824 |
| Unspecified dementia | 0.31 | 0.31 | 0.99 (0.54-1.81) | 0.975 |
| Psychiatric diseases | 4.49 | 2.88 | 1.57 (1.31-1.88) | **<0.001** |
| Depression | 1.95 | 1.10 | 1.80 (1.35-2.39) | **<0.001** |
| Anxiety disorder | 3.25 | 1.90 | 1.70 (1.37-2.12) | **<0.001** |

#### **eTable4. 12-month outcomes of PCSK9i versus statins in patients with ischemic heart disease**

| Outcome | Risk % (PCSK9i) | Risk % (Statin) | HR (95 CI) | p (Log-rank) |
| --- | --- | --- | --- | --- |
| MACE | 7.69 | 9.21 | 0.82 (0.78-0.87) | **<0.001** |
| Death | 1.78 | 4.39 | 0.40 (0.37-0.43) | **<0.001** |
| Stroke | 1.41 | 1.47 | 0.94 (0.85-1.05) | 0.305 |
| AMI | 5.25 | 5.71 | 0.74 (0.71-0.77) | **<0.001** |
| HF | 4.82 | 5.97 | 0.79 (0.75-0.84) | **<0.001** |
| dementia | 0.30 | 0.38 | 0.78 (0.63-0.97) | 0.028 |
| Vascular dementia | 0.07 | 0.06 | 1.02 (0.62-1.68) | 0.943 |
| Dementia in other diseases | 0.09 | 0.07 | 1.21 (0.77-1.89) | 0.411 |
| Unspecified dementia | 0.22 | 0.28 | 0.77 (0.60-1.00) | 0.047 |
| Parkinson’s disease | 0.10 | 0.12 | 0.84 (0.57-1.23) | 0.367 |
| Neurodegenerative diseases | 0.39 | 0.48 | 0.80 (0.66-0.97) | 0.023 |
| Psychiatric diseases | 3.20 | 2.40 | 1.28 (1.19-1.37) | 0.010 |
| Psychosis | 0.06 | 0.06 | 0.99 (0.59-1.65) | 0.970 |
| Bipolar disorder | 0.05 | 0.04 | 1.41 (0.76-2.61) | 0.270 |
| Depression | 1.34 | 1.05 | 1.27 (1.13-1.43) | **<0.001** |
| Anxiety disorder | 2.64 | 1.96 | 1.34 (1.23-1.46) | **<0.001** |

#### **eTable5. 12-month outcomes of PCSK9i versus statins in patients with Cerebrovascular disease**

| Outcome | Risk % (PCSK9i) | Risk % (Statin) | HR (95 CI) | p (Log-rank) |
| --- | --- | --- | --- | --- |
| MACE | 9.79 | 12.59 | 0.76 (0.69-0.83) | **<0.001** |
| Death | 3.11 | 7.15 | 0.42 (0.38-0.47) | **<0.001** |
| Stroke | 3.24 | 4.14 | 0.77 (0.67-0.88) | **<0.001** |
| AMI | 4.82 | 5.14 | 0.88 (0.81-0.94) | 0.001 |
| HF | 5.43 | 5.85 | 0.92 (0.83-1.01) | 0.093 |
| Neurodegenerative diseases | 1.10 | 1.38 | 0.79 (0.64-0.96) | 0.018 |
| dementia | 0.94 | 1.23 | 0.76 (0.61-0.94) | 0.011 |
| Vascular dementia | 0.28 | 0.20 | 1.37 (0.86-2.16) | 0.181 |
| Dementia in other diseases | 0.34 | 0.31 | 1.06 (0.72-1.56) | 0.765 |
| Unspecified dementia | 0.59 | 0.84 | 0.69 (0.53-0.90) | 0.006 |
| Alzheimer’s disease | 0.27 | 0.23 | 1.12 (0.72-1.74) | 0.605 |
| Parkinson’s disease | 0.22 | 0.21 | 1.03 (0.64-1.67) | 0.893 |
| Psychiatric diseases | 5.46 | 4.91 | 1.10 (1.00-1.22) | 0.046 |
| Psychosis | 0.08 | 0.11 | 0.67 (0.32-1.40) | 0.279 |
| Bipolar disorder | 0.07 | 0.06 | 1.02 (0.62-1.68) | 0.943 |
| Depression | 2.13 | 2.27 | 0.94 (0.81-1.09) | 0.411 |
| Anxiety disorder | 4.10 | 3.72 | 1.09 (0.98-1.22) | 0.123 |

**eTable6. 12-month outcomes of PCSK9i versus statins in patients with peripheral arterial disease**

| Outcome | Risk % (PCSK9i) | Risk % (Statin) | HR (95 CI) | p (Log-rank) |
| --- | --- | --- | --- | --- |
| MACE | 9.52 | 11.37 | 0.82 (0.75-0.90) | **<0.001** |
| Death | 2.79 | 5.47 | 0.50 (0.44-0.56) | **<0.001** |
| Stroke | 2.05 | 2.17 | 0.93 (0.79-1.10) | 0.391 |
| AMI | 5.34 | 5.65 | 0.97 (0.90-1.04) | 0.395 |
| HF | 6.10 | 6.97 | 0.86 (0.78-0.95) | 0.004 |
| Neurodegenerative diseases | 0.72 | 0.67 | 1.05 (0.80-1.37) | 0.728 |
| dementia | 0.60 | 0.58 | 1.02 (0.76-1.37) | 0.903 |
| Vascular dementia | 0.20 | 0.14 | 1.38 (0.79-2.41) | 0.254 |
| Dementia in other diseases | 0.16 | 0.16 | 0.97 (0.55-1.70) | 0.909 |
| Unspecified dementia | 0.36 | 0.46 | 0.78 (0.54-1.12) | 0.170 |
| Alzheimer’s disease | 0.12 | 0.16 | 0.76 (0.41-1.40) | 0.374 |
| Parkinson’s disease | 0.17 | 0.13 | 1.32 (0.73-2.38) | 0.358 |
| Psychiatric diseases | 5.25 | 4.04 | 1.30 (1.16-1.44) | **<0.001** |
| Psychosis | 0.12 | 0.10 | 1.22 (0.62-2.40) | 0.561 |
| Bipolar disorder | 0.07 | 0.06 | 1.02 (0.62-1.68) | 0.943 |
| Depression | 2.04 | 1.55 | 1.31 (1.11-1.56) | 0.002 |
| Anxiety disorder | 3.91 | 2.79 | 1.39 (1.23-1.58) | **<0.001** |

#### **eTable7. 12-month outcomes of PCSK9i–statin combination therapy versus statin therapy in patients with ASCVD**

| Outcome | Risk % (PCSK9i) | Risk % (Statin) | HR (95 CI) | p (Log-rank) |
| --- | --- | --- | --- | --- |
| MACE | 8.15 | 7.90 | 1.02 (0.88-1.19) | 0.793 |
| Death | 2.33 | 3.40 | 0.66 (0.55-0.80) | **<0.001** |
| Stroke | 1.50 | 1.25 | 1.17 (0.88-1.56) | 0.289 |
| AMI | 4.39 | 4.77 | 0.94 (0.86-1.03) | 0.192 |
| HF | 4.79 | 5.10 | 0.92 (0.78-1.08) | 0.294 |
| Neurodegenerative diseases | 0.36 | 0.21 | 1.62 (0.88-3.02) | 0.120 |
| dementia | 0.32 | 0.17 | 1.78 (0.90-3.49) | 0.091 |
| Psychiatric diseases | 3.10 | 1.95 | 1.55 (1.26-1.91) | **<0.001** |
| Depression | 1.31 | 0.83 | 1.53 (1.11-2.10) | 0.009 |
| Anxiety disorder | 2.18 | 1.53 | 1.37 (1.08-1.74) | 0.009 |

#### **eTable8. 12-month outcomes of PCSK9i monotherapy versus statin therapy in patients with ASCVD**

| Outcome | Risk % (PCSK9i) | Risk % (Statin) | HR (95 CI) | p (Log-rank) |
| --- | --- | --- | --- | --- |
| MACE | 6.76 | 8.75 | 0.76 (0.72-0.81) | **<0.001** |
| Death | 1.59 | 3.89 | 0.39 (0.36-0.42) | **<0.001** |
| Stroke | 1.29 | 1.54 | 0.82 (0.74-0.91) | **<0.001** |
| AMI | 4.62 | 5.49 | 0.75 (0.72-0.78) | **<0.001** |
| HF | 4.08 | 4.88 | 0.82 (0.77-0.87) | **<0.001** |
| Neurodegenerative diseases | 0.37 | 0.42 | 0.83 (0.69-1.00) | 0.054 |
| dementia | 0.28 | 0.35 | 0.77 (0.62-0.95) | 0.013 |
| Vascular dementia | 0.06 | 0.07 | 0.84 (0.52-1.34) | 0.460 |
| Dementia in other diseases | 0.10 | 0.09 | 1.15 (0.78-1.68) | 0.486 |
| Unspecified dementia | 0.20 | 0.20 | 0.97 (0.74-1.26) | 0.798 |
| Alzheimer’s disease | 0.09 | 0.08 | 1.05 (0.70-1.58) | 0.806 |
| Parkinson’s disease | 0.10 | 0.08 | 1.26 (0.85-1.87) | 0.243 |
| Psychiatric diseases | 3.36 | 2.42 | 1.35 (1.26-1.45) | **<0.001** |
| Psychosis | 0.05 | 0.05 | 0.96 (0.58-1.59) | 0.872 |
| Bipolar disorder | 0.05 | 0.04 | 1.08 (0.62-1.88) | 0.776 |
| Depression | 1.25 | 0.97 | 1.24 (1.10-1.38) | **<0.001** |
| Anxiety disorder | 2.52 | 1.76 | 1.38 (1.27-1.49) | **<0.001** |

**eTable9. 12-month outcomes of PCSK9i versus statin** **plus ezetimibe or bempedoic acid therapy in patients with ASCVD**

| Outcome | Risk % (PCSK9i) | Risk % (Statin) | HR (95 CI) | p (Log-rank) |
| --- | --- | --- | --- | --- |
| MACE | 7.35 | 7.29 | 1.00 (0.95-1.05) | 0.894 |
| Death | 1.70 | 2.83 | 0.59 (0.54-0.63) | **<0.001** |
| Stroke | 1.40 | 1.33 | 1.03 (0.93-1.14) | 0.588 |
| AMI | 5.63 | 5.98 | 0.93 (0.89-0.97) | **<0.001** |
| HF | 4.43 | 4.42 | 0.98 (0.92-1.04) | 0.524 |
| Neurodegenerative diseases | 0.41 | 0.38 | 1.04 (0.87-1.25) | 0.640 |
| dementia | 0.31 | 0.31 | 0.99 (0.81-1.21) | 0.907 |
| Vascular dementia | 0.06 | 0.06 | 1.00 (0.63-1.58) | 0.997 |
| Dementia in other diseases | 0.10 | 0.10 | 1.04 (0.73-1.48) | 0.836 |
| Unspecified dementia | 0.21 | 0.22 | 0.95 (0.74-1.21) | 0.679 |
| Alzheimer’s disease | 0.08 | 0.08 | 0.97 (0.66-1.44) | 0.886 |
| Parkinson’s disease | 0.11 | 0.08 | 1.32 (0.92-1.90) | 0.133 |
| Psychiatric diseases | 3.33 | 2.34 | 1.39 (1.30-1.49) | **<0.001** |
| Psychosis | 0.06 | 0.04 | 1.32 (0.79-2.22) | 0.287 |
| Bipolar disorder | 0.05 | 0.04 | 1.09 (0.64-1.85) | 0.752 |
| Depression | 1.29 | 0.87 | 1.44 (1.29-1.61) | **<0.001** |
| Anxiety disorder | 2.46 | 1.67 | 1.44 (1.33-1.56) | **<0.001** |

#### **eTable10. 24-month outcomes of PCSK9i versus statins in patients with ASCVD**

| Outcome | Risk % (PCSK9i) | Risk % (Statin) | HR (95 CI) | p (Log-rank) |
| --- | --- | --- | --- | --- |
| MACE | 13.06 | 14.00 | 0.90 (0.87-0.94) | **<0.001** |
| Death | 3.48 | 6.50 | 0.50 (0.47-0.53) | **<0.001** |
| Stroke | 2.64 | 2.53 | 1.01 (0.93-1.09) | 0.860 |
| AMI | 5.42 | 6.16 | 0.89 (0.86-0.92) | **<0.001** |
| HF | 7.95 | 8.17 | 0.94 (0.90-0.99) | 0.014 |
| Neurodegenerative diseases | 0.85 | 0.85 | 0.96 (0.84-1.10) | 0.585 |
| dementia | 0.67 | 0.70 | 0.91 (0.78-1.06) | 0.248 |
| Vascular dementia | 0.16 | 0.12 | 1.33 (0.96-1.85) | 0.088 |
| Dementia in other diseases | 0.21 | 0.23 | 0.93 (0.72-1.20) | 0.578 |
| Unspecified dementia | 0.46 | 0.50 | 0.88 (0.74-1.05) | 0.146 |
| Alzheimer’s disease | 0.18 | 0.19 | 0.93 (0.71-1.23) | 0.614 |
| Parkinson’s disease | 0.22 | 0.18 | 1.22 (0.92-1.62) | 0.162 |
| Psychiatric diseases | 6.63 | 4.72 | 1.39 (1.32-1.46) | **<0.001** |
| Psychosis | 0.10 | 0.11 | 0.87 (0.59-1.27) | 0.457 |
| Bipolar disorder | 0.09 | 0.08 | 1.15 (0.75-1.78) | 0.518 |
| Depression | 2.54 | 1.69 | 1.49 (1.36-1.62) | **<0.001** |
| Anxiety disorder | 4.93 | 3.33 | 1.47 (1.38-1.57) | **<0.001** |

**eTable11. 36-month outcomes of PCSK9i versus statins in patients with ASCVD**

| Outcome | Risk % (PCSK9i) | Risk % (Statin) | HR (95 CI) | p (Log-rank) |
| --- | --- | --- | --- | --- |
| MACE | 17.92 | 18.65 | 0.92 (0.89-0.96) | **<0.001** |
| Death | 5.24 | 8.77 | 0.54 (0.52-0.57) | **<0.001** |
| Stroke | 3.58 | 3.39 | 1.02 (0.94-1.09) | 0.697 |
| AMI | 7.28 | 8.45 | 0.94 (0.91-0.97) | 0.001 |
| HF | 11.02 | 10.50 | 1.01 (0.96-1.05) | 0.737 |
| Neurodegenerative diseases | 1.38 | 1.26 | 1.04 (0.92-1.17) | 0.530 |
| dementia | 1.15 | 1.03 | 1.04 (0.91-1.18) | 0.585 |
| Vascular dementia | 0.25 | 0.20 | 1.20 (0.89-1.61) | 0.224 |
| Dementia in other diseases | 0.38 | 0.33 | 1.06 (0.84-1.34) | 0.598 |
| Unspecified dementia | 0.77 | 0.76 | 0.97 (0.83-1.14) | 0.749 |
| Alzheimer’s disease | 0.32 | 0.28 | 1.09 (0.85-1.41) | 0.490 |
| Parkinson’s disease | 0.32 | 0.29 | 1.13 (0.88-1.45) | 0.328 |
| Psychiatric diseases | 9.18 | 6.36 | 1.43 (1.37-1.51) | **<0.001** |
| Psychosis | 0.13 | 0.14 | 0.88 (0.62-1.26) | 0.488 |
| Bipolar disorder | 0.13 | 0.11 | 1.10 (0.75-1.63) | 0.616 |
| Depression | 3.55 | 2.32 | 1.52 (1.40-1.64) | **<0.001** |
| Anxiety disorder | 7.05 | 4.62 | 1.51 (1.42-1.59) | **<0.001** |

**eTable12. 12-month negative control outcomes of PCSK9i versus statins in patients with ASCVD**

| Outcome | Risk % (PCSK9i) | Risk % (Statin) | HR (95 CI) | p (Log-rank) |
| --- | --- | --- | --- | --- |
| Composite NCO | 5.61 | 5.49 | 1.02 (0.97–1.08) | 0.426 |
| Otitis externa | 1.20 | 1.17 | 1.03 (0.92–1.15) | 0.589 |
| Folliculitis | 1.01 | 1.06 | 0.95 (0.85–1.07) | 0.391 |
| Kidney Stones | 1.20 | 1.15 | 1.04 (0.93–1.16) | 0.486 |
| Gallbladder Stones | 1.24 | 1.27 | 0.97 (0.87–1.08) | 0.624 |
| Burn | 0.95 | 0.94 | 1.01 (0.89–1.15) | 0.870 |

#### **All abbreviations apply throughout the Supplementary Material.**

AF, atrial fibrillation; AMI, acute myocardial infarction; ASCVD, atherosclerotic cardiovascular disease; CAD, coronary artery disease; IHD, ischemic heart disease; CKD, chronic kidney disease; CI, confidence interval; CPT, Current Procedural Terminology; EHR, electronic health record; HR, hazard ratio; HCO, healthcare organization; HBP, hypertension; ICD-10-CM, International Classification of Diseases, 10th Revision, Clinical Modification; MACE, major adverse cardiovascular events; MI, myocardial infarction; NCO: negative control outcome; PAD, peripheral arterial disease; PCSK9i, proprotein convertase subtilisin/kexin type 9 inhibitor; PSM, propensity score matching; VA, Veterans Affairs; GDPR, General Data Protection Regulation; HIPAA, Health Insurance Portability and Accountability Act.
